# Soil microorganism distributions depend on habitat partitioning of topography in a temperate mountain forest

**DOI:** 10.1128/spectrum.02056-24

**Published:** 2025-05-28

**Authors:** Jianyou Li, Xueying Li, Shengqian Guo, Jingjing Xi, Yizhen Shao, Yun Chen, Zhiliang Yuan

**Affiliations:** 1 College of Life Science, Henan Agricultural University 70573, Zhengzhou, China; Fujian Agriculture and Forestry University, Fuzhou City, China

**Keywords:** soil microorganisms, topographic habitat, community structure, ecological specialization

## Abstract

As the main decomposers in forest ecosystems, soil microorganisms are crucial in maintaining ecosystem functions and services. Topographic factors are essential for soil formation and can influence the community structure of soil microorganisms by modifying the soil environment. However, a systematic understanding of the distribution mechanisms of soil microorganisms across different terrain habitats is still lacking. This study characterized soil bacteria and fungi in the valleys, mid-slopes, and ridges of the temperate deciduous broadleaf forest in Baiyun Mountain, Luoyang, to analyze the effect of topographic habitat on the distribution patterns of soil microbial communities. Results showed that the distribution of most soil microorganisms in different terrain habitats follows the principle of ecological specialization. The distribution patterns of soil bacteria and fungi are related to the terrain habitat, and the fungal community (60.48%) exhibits a stronger habitat specificity than the bacterial community (31.78%). Soil moisture has a greater influence on fungal communities than bacterial communities, whereas soil physical and chemical properties more significantly explain variations in bacterial community distribution. These findings indicate that topographic habitat significantly influences soil microbial community distribution, and bacteria show stronger habitat adaptability than fungi.

**IMPORTANCE:**

This study provides an in-depth examination of the impact of topographic habitat on the structural composition and spatial distribution characteristics of bacterial and fungal communities. The research focused on three distinct terrain habitats: valley, midslope, and ridge. Our results indicate that soil bacterial and fungal networks, along with major environmental factors, shape the composition and distribution of soil microbial communities across different terrain habitats. We found that fungi exhibit stronger habitat specificity than bacteria and are more likely to thrive in valleys with higher water content. Furthermore, major environmental factors significantly influence the distribution of soil microbial communities. These findings could inform the development of more effective forest soil management and conservation strategies tailored to different topographic habitats.

## INTRODUCTION

Soil microorganisms are an essential component of soil and play a crucial role in regulating ecological processes such as nutrient cycling and carbon and nitrogen cycling (1). The structure and distribution of soil microbial communities are influenced not only by the soil’s physical and chemical properties but also by the topographic characteristics. Topography controls the reception and redistribution of water, temperature, nutrients, and organic matter on the surface, affecting soil erosion, water storage, weathering, nutrient leaching, and the translocation and storage of soil organic matter (2, 3). These processes indirectly affect the community structure of soil microorganisms. For instance, soil moisture (4), nitrogen concentration (5, 6), soil pH (7), and soil carbon content (8) influence the composition and function of soil microbial communities. In addition, solar radiation and soil erosion indirectly affect the composition and activity of soil microbial communities by influencing soil temperature, water retention, and nutrient dynamics (9). Various combinations of major ecological processes drive the growth and development of microbial communities (7, 10, 11). These factors collectively determine the survival and reproduction conditions of soil microorganisms. Therefore, studying the effects of different topographic habitats on soil microorganisms is vital for understanding how topography drives soil ecological processes.

Niche theory emphasizes that the species composition of different habitats is related to the differences in the environmental requirements of species and the spatial distribution of environmental conditions (12, 13). Although ecologists have studied a variety of factors affecting soil microorganisms, including abiotic and biotic factors (14, 15), the distribution mechanisms of soil microorganisms in different habitats remain unclear. Many studies have shown that bacteria and fungi have different environmental requirements and coexistence mechanisms (16, 17). Bacteria generally have higher growth and turnover rates, whereas fungi exhibit slower growth and turnover rates (18). Compared with bacteria, soil fungi are generally more resistant but less resilient to environmental changes (19–22). The different responses of soil microorganisms to environmental conditions may influence their distribution across various habitats.

Increasing evidence suggests that the characteristics of ecological networks may represent interactions between coexisting organisms, thereby affecting community responses to environmental changes (20, 23, 24). For example, theoretical studies predict that ecological networks composed of weak interactions are more stable compared with those with strong interactions (25, 26). In addition, an increase in negative interactions improves network stability under disturbance (26–28). Therefore, communities connected primarily by positive correlations are considered unstable. In such communities, species may produce negative feedback and co-oscillations in different topographic habitats (26). Negative correlations can stabilize co-oscillations within the community and promote network stability (26). Positive correlations between fungal and bacterial communities may represent a series of interactions or simply indicate similar responses to different topographical habitats (29, 30). Although caution is required when interpreting these relationships (30, 31), symbiotic networks can provide insights into the co-oscillations and stability of microbial communities (29). Despite these studies and the increasing use of network analysis in ecology (23, 29, 32, 33), our understanding of the symbiotic or potential interactions within complex soil microbial communities and their distribution across topographic habitats remains limited.

Soil microorganisms are extremely sensitive to environmental changes, and factors such as humidity, salinity, temperature, and pH significantly alter soil microbial community structures (34). Global climate change, one of the most critical environmental challenges facing human society today, threatens ecosystem diversity and stability, significantly affecting their functions (35). While numerous studies have examined various ecosystems, including forests, farmland (36, 37), wetlands (38), and grasslands (39), notable differences are observed in how bacterial and fungal community characteristics respond to moisture changes across different topographic habitats. However, research on the distribution mechanisms of soil microorganisms within different topographic habitats in forest ecosystems remains scarce.

To understand the influence of different topographic habitats in a temperate deciduous broad-leaved forest on the community structure and distribution mechanisms of soil microorganisms, 16S rRNA and ITS segment amplicon sequencing were utilized to analyze the surface soil (0–20 cm) microbial community composition across various valley, mid-slope, and ridge forest plots. Our analysis focused on microbial community structure, diversity, and environmental influences. Specifically, the following aspects were explored: (i) whether soil microorganisms (fungi and bacteria) exhibit preferences for particular terrain habitats, (ii) how different terrains affect the spatial distribution of soil microorganisms and the structure of microbial communities, and (iii) the main environmental factors influencing the distribution of soil microbial communities across different terrains.

## MATERIALS AND METHODS

### Overview of the study area

**Fig 1:**
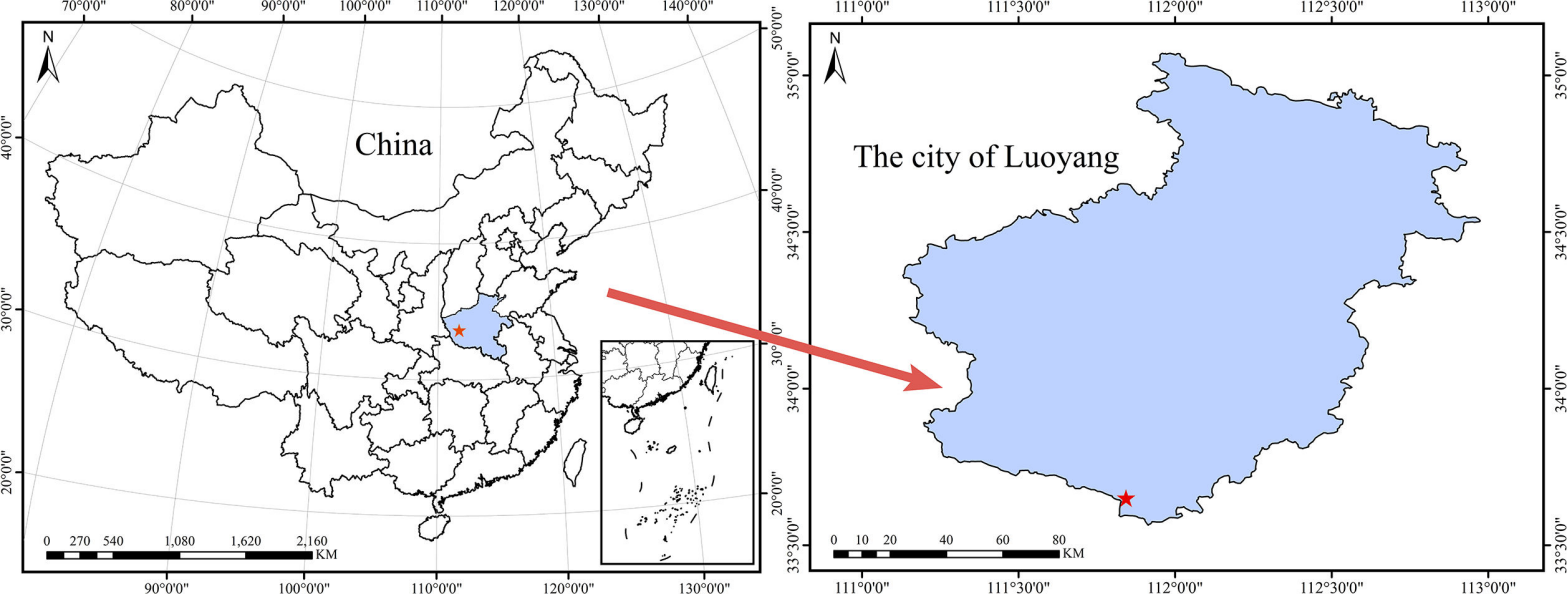
Location of the study area and sampling points. The red star indicates the location of the sample plots.

This study was conducted in Baiyun Mountain, China (between 111° 48′–112° 16′ E, 33° 33′–33° 56′ N) (Fig. 1). The region features a transitional climate between subtropical and warm temperate zones and covers an area of approximately 168 km². The average altitude is about 1,500 m, the highest peak reaches 2,216 m, and mountain slopes typically range from 40° to 80°. The average annual temperature is 18°C, the average annual precipitation is 1,200 mm, and the average relative humidity is 70%–78%.

The forest type is temperate deciduous broad-leaved forest, with a forest coverage rate of 98.5% and a total of 1,991 species of woody plants. Dominant species include *Quercus aliena Blume* var. *acuteserrata* Maxim, *Pinus armandii* Franch., *Larix gmelinii* (Rupr.) Kuzen., and *Forsythia suspensa* (Thunb.) Vahl.

### Sample site selection and soil data determination

To compare the distribution preferences of soil microorganisms in different terrain habitats, 10 quadrats of 20 m × 20 m each were established in three terrain habitats, namely, the valley, mid-slope, and ridge of Baiyun Mountain, totaling 30 quadrats. Each 20 m × 20 m quadrat served as the basic unit for sampling surface soil (0–20 cm). Three replicates were randomly sampled using a soil corer at each sampling point. After removing visible roots, plant leaves, rock fragments, and large organic debris, the three samples were combined into a composite sample. The composite soil samples were sieved to 2 mm and divided into two subsets: One subset was stored at −80°C for high-throughput sequencing, and the other subset was stored at 4°C for soil physical and chemical property determination.

The indicators selected for the measurement of soil physical and chemical properties include soil pH, soil moisture content (SWC), soil organic matter (SOM), soil available phosphorus (P), and soil alkali-hydrolyzable nitrogen (N). Soil pH was measured using a glass electrode meter (InsMark IS126, Shanghai, China) with a water-to-soil ratio of 2.5:1. SWC was determined by oven-drying fresh samples (∼10 g) at 105°C to a constant weight (40). SOM was measured using the potassium dichromate volumetric method and the external heating method, and the results were multiplied by a correction factor of 1.1 (41). Soil available phosphorus (P) was determined using the 0.5 M NaHCO₃ extraction method and the molybdenum antimony colorimetric method (42). Soil alkali-hydrolyzable nitrogen (N) was measured using the alkaline hydrolysis diffusion method (41).

### DNA extraction, PCR amplification, and high-throughput sequencing

The total DNA of the microbial communities was extracted from the soil samples using the FastDNA Spin Kit for Soil (MP Biomedicals, Norcross, GA, USA). The quality of the extracted DNA was verified by 1% agarose gel electrophoresis, and the concentration and purity were measured using a NanoDrop 2000 spectrophotometer. The bacterial 16S rRNA gene V3-V4 variable region was PCR amplified using primers 515F (5ʹ-GTGCCAGCMGCCGCGGTAA-3ʹ) and 806R (5ʹ-GGACTACHVGGGTWTCTAAT-3ʹ) (43), and the fungal ITS segment was PCR amplified using primers ITS1F (5ʹ-CTTGGTCATTTAGAGGAAGTAA-3ʹ) and ITS2R (5ʹ-GCTGCGTTCTTCATCGATGC-3ʹ). Each sample was amplified in triplicate. After mixing the PCR products, they were recovered using a 2% agarose gel, purified using the AxyPrep DNA Gel Extraction Kit (Axygen Biosciences, Union City, CA, USA), and quantified with a Quantus Fluorometer (Promega, USA (44);). Libraries were prepared using the NEXTFLEX Rapid DNA-Seq Kit and sequenced on the Illumina MiSeq PE300 platform (Shanghai Meiji Biopharmaceutical Technology Co., Ltd.). After quality trimming at a threshold of an average quality score >20, sequences were clustered into operational taxonomic units (OTUs) based on 97% similarity using UPARSE software (Version 7.1; http://drive5.com/uparse/ [45]). Chimeras were removed using UCHIME software. Finally, sequences were annotated with species classification using the RDP classifier (http://rdp.cme.msu.edu/) and aligned to the Silva database (SSU128) with a 70% alignment threshold.

### Environmental data collection

The elevation of each small quadrat was measured using a total station (RTK), and the slope, aspect, and concavity were calculated based on the elevation data (46, 47). Concurrently, light data within the forest were collected during soil microorganism investigations using an SLM9-UM-1.2 canopy analyzer (Delta-T Devices Co, Ltd.). For each 20 m × 20 m quadrat, the lighting data represent the average values obtained from its four smaller quadrats. These measurements include light transmittance (LT), scattered radiation (SR), total radiation (TR), canopy coverage (CC), average leaf angle (ALA), and leaf area index (LAI).

### Statistical analysis

The non-metric multidimensional scaling (NMDS) analysis was performed on the Bray-Curtis dissimilarities. This analysis utilized the “metaMDS” function in the “vegan” package, and community centroids were fitted onto the NMDS plot using the “envfit” function. One-way ANOVA was conducted to compare microbial community species composition across different terrains, employing the “aov” function from the “stats” package.

To visualize the relationship between OTUs and terrains, a chord diagram was generated using the “ChordDiagram” function from the “circlize” package. This diagram illustrates the composition of dominant species across various habitat types. In addition, a ternary plot was created using the “ggtern” package to depict the enrichment patterns of bacterial and fungal OTUs across varying terrain habitats, thereby elucidating the distribution preferences of each community along moisture gradients.

A symbiotic network analysis was utilized to assess the habitat specificity of the fungi and bacteria across the different terrains. Results from this network analysis were visualized using Gephi software (48), and the community-microbial network structure was evaluated using the modularity index (49). Torus transformation was applied to assess the correlation between microorganisms in Baiyun Mountain, which included 15,555 OTUs for bacteria and 5,250 OTUs for fungi, focusing on positive correlations (*P* ≤ 0.05). This analysis also accounted for spatial autocorrelation of species distribution (46). Before analysis, OTUs with relative abundances less than 0.01% were excluded to minimize the effects of rare taxa.

Redundancy analysis (RDA) was employed to investigate the influence of environmental factors on soil microbial communities, using the “vegan” package in R. Environmental factors influencing microbial species composition were categorized into soil physical and chemical properties (pH, P, N, SWC, and SOM), terrain factors (aspect, slope, elevation, and concavity), and light parameters (LT, SR, TR, CC, LAI, and ALA). Linear discriminant analysis effect size (LEfSe) was used to identify significantly different microorganisms in the forest soils across various terrain habitats, and a linear discriminant analysis (LDA) threshold was set at 2 (50).

Furthermore, a structural equation model (SEM) was constructed using the “PiecewiseSEM” package to explore the direct and indirect effects of significant influencing factors (*P* < 0.01) on bacteria and fungi across different terrain habitats. All statistical analyses were conducted using R software (version 4.3.2).

## RESULTS

### Composition of soil microorganisms in different terrain habitats is different

**Fig 2:**
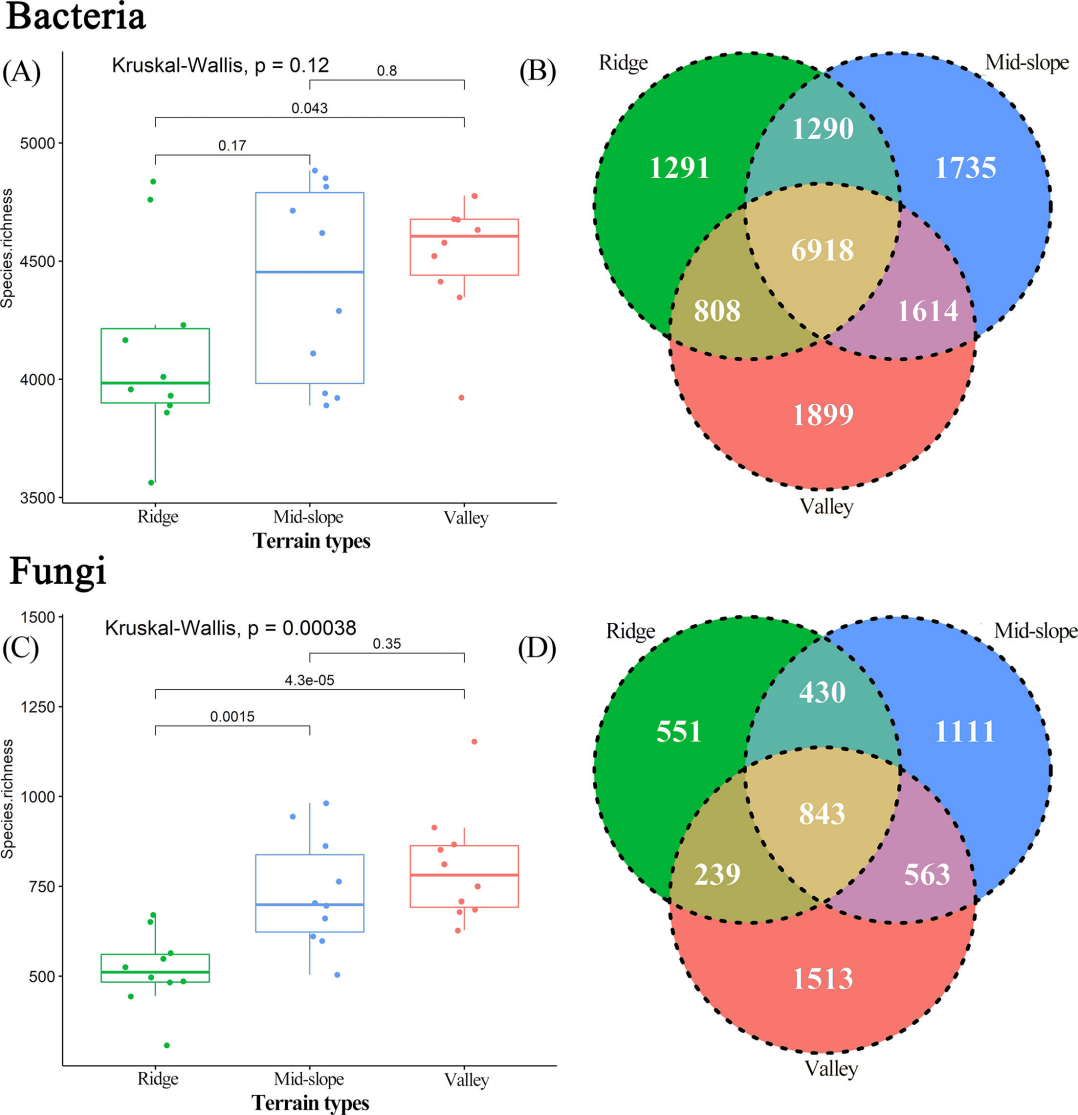
Box plots ([A] bacteria; [C] fungi) show the differences in soil microbial OTU richness across different terrain habitats. Venn diagrams ([B] bacteria; [D] fungi) illustrate the number of unique and shared OTUs in the different terrain habitats. Assorted color types are used to distinguish microbial communities in the three terrain habitats.

**Fig 3:**
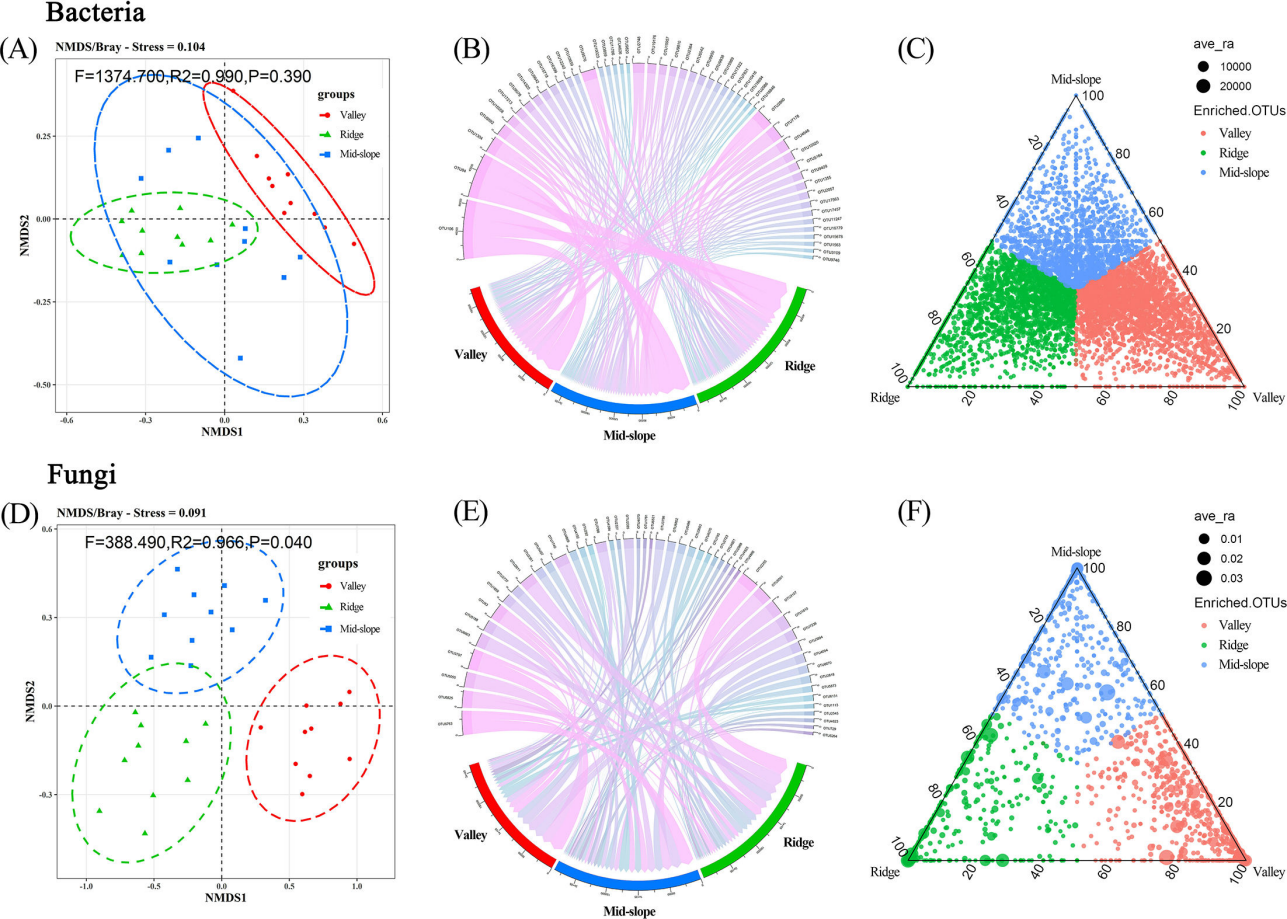
NMDS analysis ([A] bacteria; [D] fungi) investigates the species composition of microbial communities across three terrain habitats, with ellipses representing 95% confidence intervals. Chord plots ([B] bacteria; [E] fungi) display the dominant species of bacterial and fungal communities in different terrain habitats. Ternary plots ([C] bacteria; [F] fungi) reflect the enrichment of bacterial and fungal OTUs under different humidity conditions. The three vertices represent three different terrain habitats, and each point represents an OTU; point size indicates the relative abundance of each OTU, with points closer to a vertex representing a higher relative abundance in that habitat. Different colors distinguish microbial communities across the three terrain habitats.

The Kruskal–Wallis test results revealed significant differences in the OTU richness of soil microorganisms across various terrain habitats, and the species richness of bacteria and fungi decreased as terrain altitude increased (Fig. 2A and C). Fungi exhibited stronger habitat specificity; 60.48% (3,175 species) of fungal OTUs were found exclusively within a single terrain habitat compared with only 31.78% (4,943 species) of bacteria showing this specificity (Fig. 2B and D). As the number of sequences measured increased, the rarefaction curve gradually flattened, indicating sufficient sample sequences and reasonable sequencing depth (S 1). NMDS analysis showed a minor difference in the diversity distribution of bacterial OTUs among different terrain habitats (R² =0.990, *P* = 0.309; Fig. 3A), whereas a significant difference was noted in the diversity distribution of fungal OTUs (R² =0.966, *P* = 0.040; Fig. 3D). Although there were significant differences in species composition among different terrain habitats, the composition of dominant microbial species was similar (Fig. 3B and E). The ternary plot results show that fungal OTUs are mainly enriched in the valley habitat and have higher relative abundance in this habitat (Fig. 3D). By contrast, the distribution of bacteria across different terrain habitats is less distinct than that of fungi (Fig. 3C).

Using LEfSe analysis, microorganisms exhibiting significant differences between different terrain habitat groups (LDA threshold = 2) were identified (S2). Notably, among bacteria showing significant differences, Gaiellales, Methyloligellaceae, and Pseudolabrys were more abundant in the valley group, whereas Paraburkholderia, Mycobacterium, and Xanthobacterium were more abundant in the mid-slope group. By contrast, norank, Bradyrhizobium, and AD3 were more prevalent in the ridge group. Among fungi, species such as elongata, Ascomycota, and Rozellomycota were more abundant in the valley group, whereas Saitozyma, Hyaloscyphaceae, and Metarhizium were more abundant in the mid-slope group. In addition, Tricholoma, Piloderma, and Helotiaceae exhibited higher abundance in the ridge group (S2).

### Spatial distribution of fungi and bacteria in different terrain habitats

**Fig 4:**
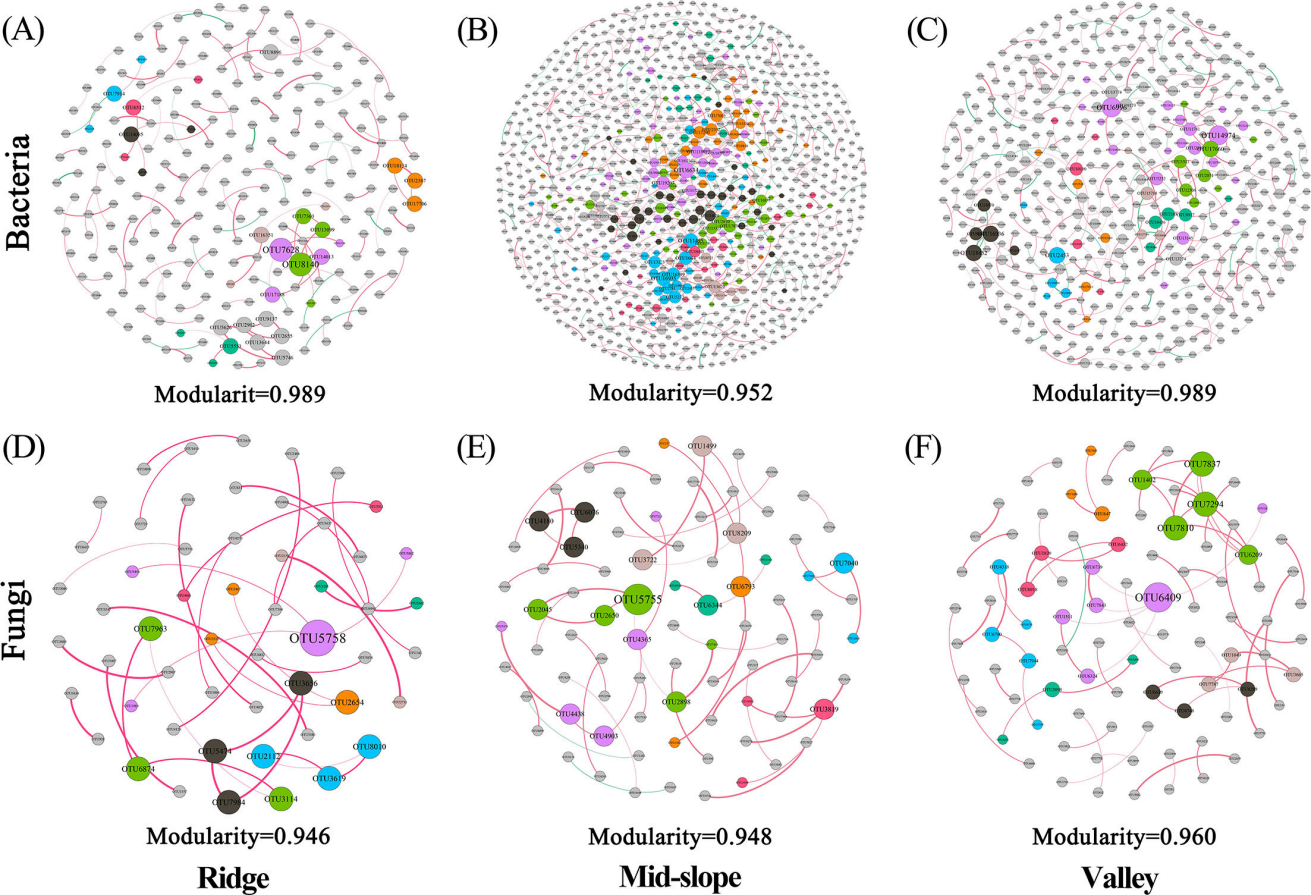
Network diagrams ([A–C] bacteria; [D–F] fungi) reveal the correlations between soil fungal and bacterial OTU modules in ridge, mid-slope, and valley habitats. Node colors indicate the module classifications of the OTUs, and edges represent strong (SparCC |r| > 0.7) and significant (*P* < 0.01) correlations. Edge thickness is proportional to the correlation coefficient, with red indicating positive correlations. Stronger positive correlations imply lower network stability. Node size is proportional to the degree (number of connections) of each OTU.

In the symbiotic network analysis, the positive correlation strength between the OTU modules of bacteria in valley and ridge habitats was higher than in mid-slope habitats (Fig. 4A through C). The positive correlation strength among the OTU modules of the fungal network was generally higher than that of bacteria. Notably, the strength of positive correlations between fungal OTU modules was highest in ridge habitats and lowest in valley habitats (Fig. 4D through F). These results indicate that the stability of fungal network modules was lower than that of bacterial modules, and the lowest stability was observed in ridge habitats.

**Fig 5:**
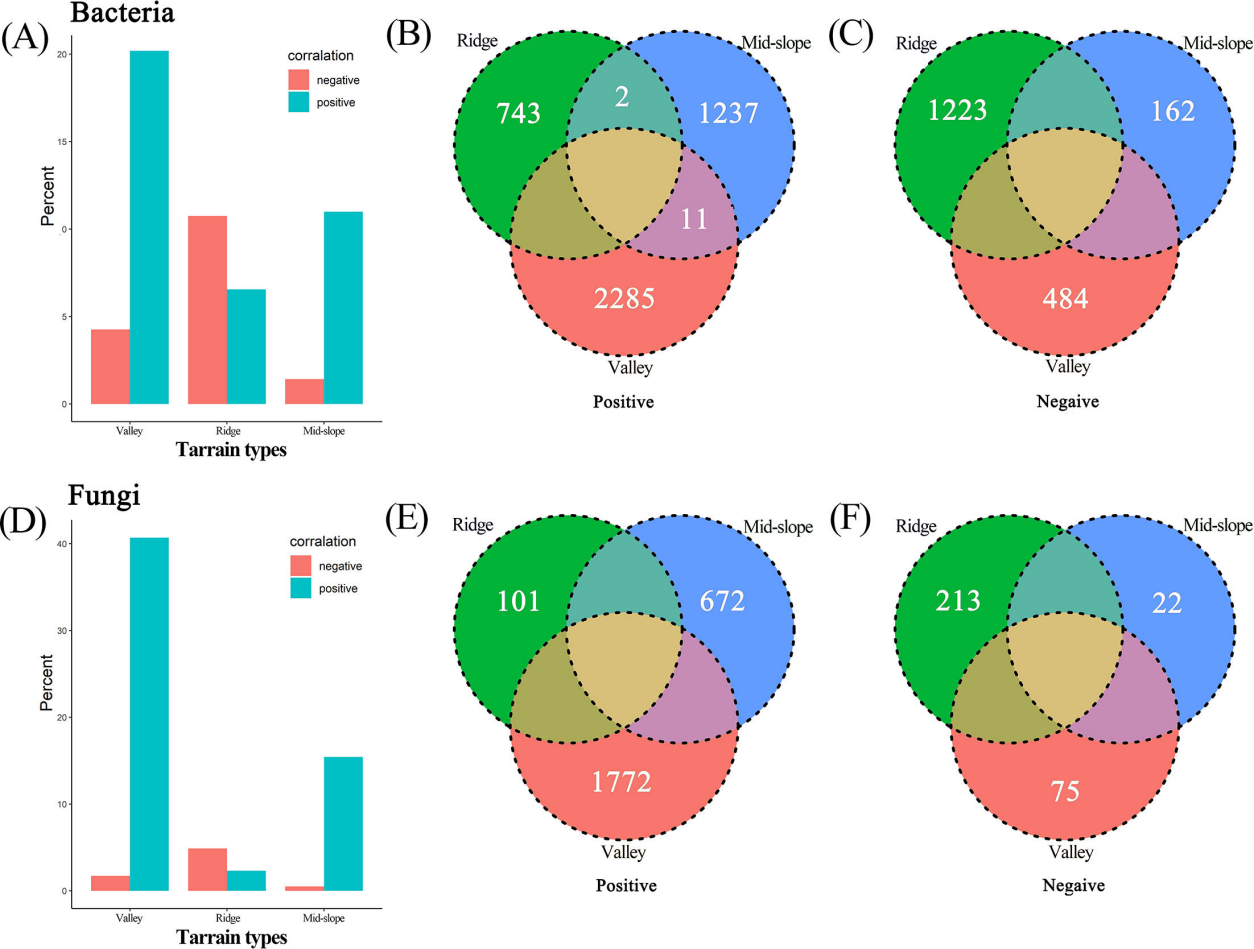
Bar graphs ([A] bacteria; [D] fungi) show the percentages of bacteria and fungi associated with different topographic habitats. Venn diagrams ([B, C] bacteria; [E, F] fungi) display the number of unique and shared species in different topographic habitats, with significant positive ([B] bacteria; [E] fungi) and negative ([C] bacteria; [F] fungi) correlations. The correlation between microbial and plant communities is evaluated using Torus-translation randomization (Torus-translation test, *P* ≤ 0.05 significance level). Different colors are used to distinguish microbial communities in the three topographic habitats.

Based on the Torus transformation test, among the 15,727 species of bacteria and fungi, 6,147 species of bacteria significantly related to the three terrain habitats accounted for 54.05% (Fig. 5A), which was lower than the 2,855 species of fungi (65.57%; Fig. 5D). Among them, 4,378 species of bacteria (37.62%) and 2,545 species of fungi (58.45) were positively correlated with terrain habitat (Fig. 5B and E). Most soil microorganisms were distributed in valleys, including 2,780 species (45.13%) of bacteria and 1,847 species (64.70%) of fungi (Fig. 5A and D).

Comparing the number of cross-correlated OTUs between different terrains (Fig. 5B, C, E, and F), no OTUs were found positively or negatively correlated with the three terrain habitats at the same time (all positive/negative correlation cross-numbers were 0). Most species are neutral to other terrain habitats, that is, they do not completely exclude the other two terrain habitats.

### Effects of environmental factors on soil microbial communities

**Fig 6:**
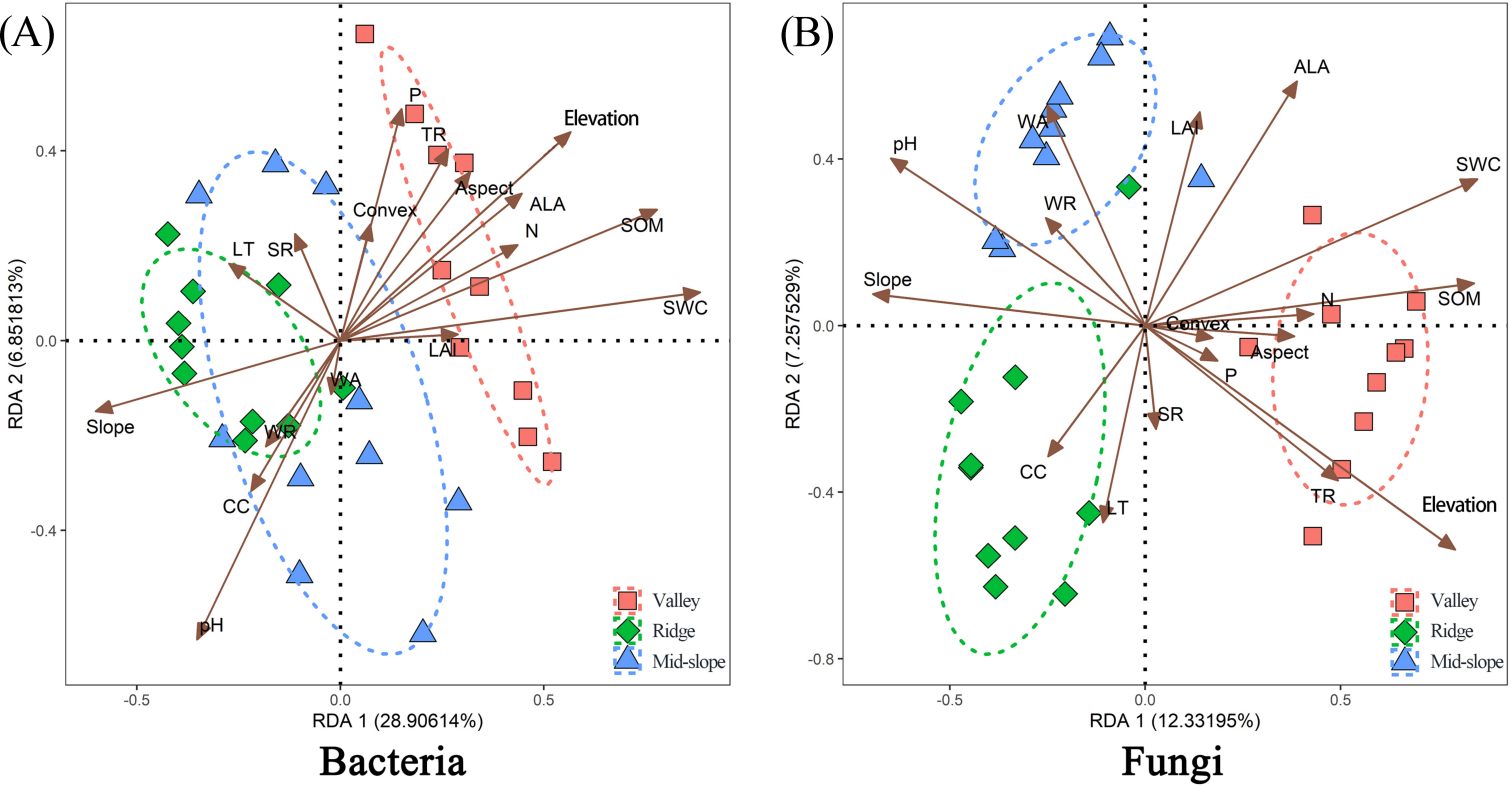
Redundancy analysis ([A] bacteria; [B] fungi) of the composition of soil microbial communities and environmental variables in various terrain habitats. Brown arrows represent environmental factors; red, green, and blue shapes, respectively, represent the composition of microbial communities in valley, ridge, and mid-slope habitats. Ellipses indicate 95% confidence intervals. Abbreviations: Elevation: elevation; Slope: slope; Aspect: slope aspect; Concavity: concavity; WA: species abundance; WR: species richness; LT: light transmittance; SR: diffuse radiation; TR: total radiation; CC: canopy cover area; ALA: average leaf angle; LAI: leaf area index; pH: soil acidity; SWC: soil water content; SOM: soil organic matter; P: soil available phosphorus; N: soil alkaline nitrogen.

Environmental factors explained 62.30% of the changes in bacterial species distribution. Among them, soil physical and chemical properties (pH, SWC, SOM, and N), terrain factors (slope and elevation), and light (ALA) had a greater influence on the distribution of bacterial communities (Fig. 6A; Table 1). The explanation degree of environmental factors on changes in the distribution of fungal species was 61.45%, which is similar to that of bacteria, and the main environmental influencing factors are also similar. The difference is that the changes in fungal species distribution were also affected by aspect, WA, and TR, and were not significantly affected by ALA (Fig. 6B; Table 1). Furthermore, soil physical and chemical properties (pH, SWC, SOM, and N) explained the changes in bacterial community distribution more than fungal communities (Table 1).

**TABLE 1:** Significance test of environmental factors influencing bacteria and fungi^*a*^

Species	Environmental factors	Pr (> r）	R^2^	Species	Environmental factors	Pr (> r）	R^2^
Bacteria	pH	0.01**	0.498	Fungi	pH	0.02*	0.234
	SWC	0.01**	0.785		SWC	0.01**	0.442
	P	0.30			P	0.42	
	SOM	0.01**	0.651		SOM	0.01**	0.491
	N	0.05*	0.203		N	0.01**	0.318
	Aspect	0.20			Aspect	0.01**	0.298
	Slope	0.01**	0.403		Slope	0.03*	0.322
	Elevation	0.01**	0.597		Elevation	0.01**	0.623
	Concavity	0.48			Concavity	0.08	
	WA	0.50			WA	0.01**	0.315
	WR	0.72			WR	0.47	
	LT	0.45			LT	0.14	
	SR	0.79			SR	0.13	
	TR	0.06			TR	0.01**	0.344
	CC	0.51			CC	0.47	
	LAI	0.57			LAI	0.31	
	ALA	0.05*	0.165		ALA	0.71	

^*a*^ *P*-values and R² values correspond to the RDA components in Fig. 6A and B, indicating the significance and explanatory power of each environmental factor. Significance levels are denoted by *P < 0.05, **P < 0.01.

**Fig 7:**
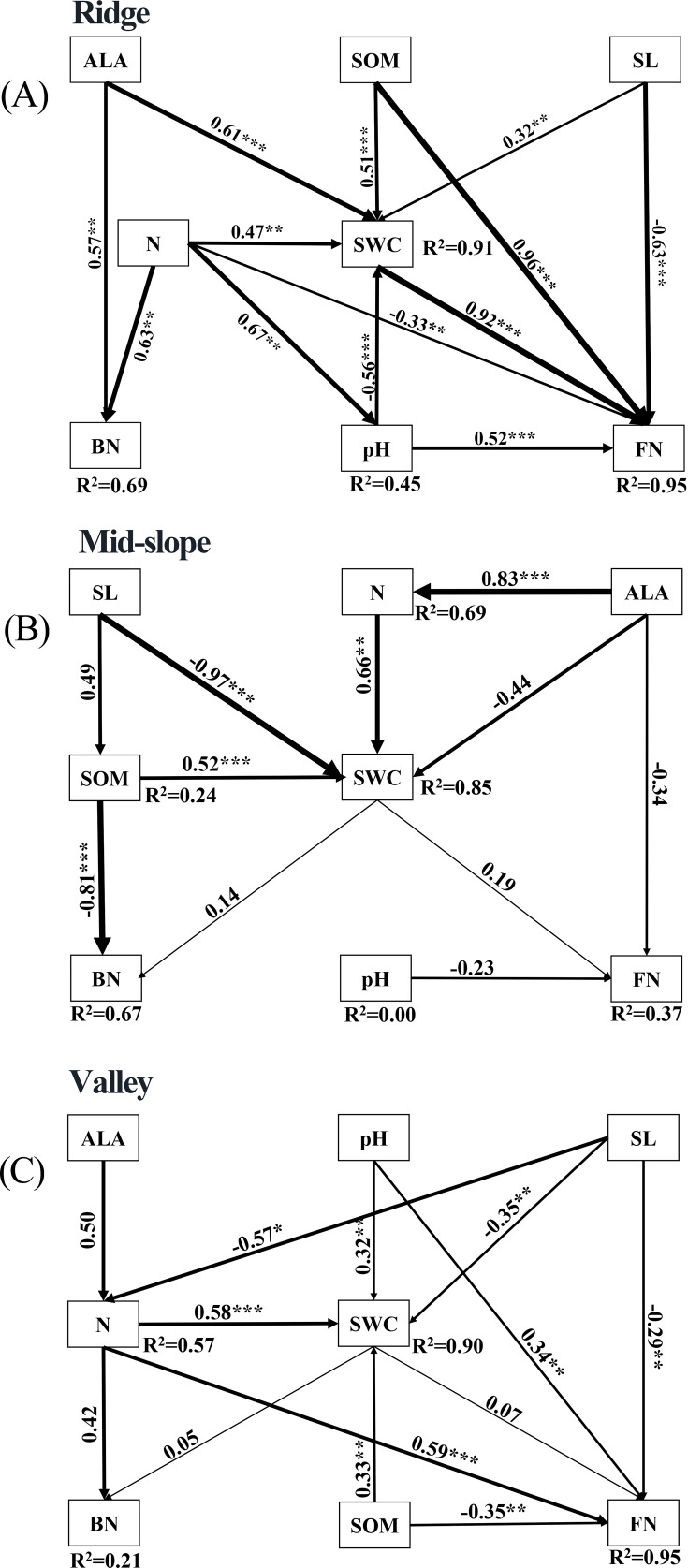
SEM ([A] ridge; [B] mid-slope; [C] valley) reveals the direct or indirect effects of moisture on soil bacteria and fungi. The number of neighbors in the same direction as the arrow indicates the path coefficient, and the width of the arrow is proportional to significance. BN and FN represent bacterial and fungal communities, respectively, while SL indicates the slope. The R² value reflects the proportion of variance explained by each variable. Significance levels are denoted by *P < 0.05, **P < 0.01, ***P < 0.001.

To elucidate the influences of environmental factors further, particularly water content in diverse topographic habitats, on the composition and distribution of soil microorganisms, SEMs were employed to investigate the direct or indirect effects of water content on the composition and distribution of bacterial and fungal species (Fig. 7A through C). The findings indicated that SWC exerted significant direct effects on fungi in the ridge habitat (Fig. 7A). Moreover, ALA, SOM, pH, N, and slope indirectly influenced the composition and distribution of fungi via SWC. Nevertheless, SWC had no notable effect on bacteria. In the mid-slope and valley habitats, SWC did not have significant effects on either bacteria or fungi (Fig. 7B and C). The results demonstrate that SWC had a greater effect on fungi than on bacteria in different topographic habitats.

## DISCUSSION

Our results show that in different topographic habitats of temperate deciduous broadleaf forests, fungi tend to be distributed in valleys with high moisture content, whereas bacteria are less influenced by topographic habitats. In addition, the relationship between bacteria and fungi and environmental factors differs in diverse terrain habitats. Overall, these findings indicate that different topographic habitats have an important effect on the structure and composition of soil microbial communities.

### Effects of topographic habitat on soil microbial composition

The species composition of bacteria and fungi varies across different terrain habitats, yet bacterial and fungal OTU richness decrease with higher altitude. This trend may be attributed to higher soil moisture content in valleys, where dense forest vegetation and abundant litter provide ample water and nutrient resources for soil microorganisms, fostering favorable growth conditions and reproductive rates (51). Precipitation also plays a crucial role in maintaining soil moisture levels (52). Valleys, being low-lying areas, effectively retain water, resulting in comparatively higher soil moisture content. Elevated humidity lowers soil temperature and enhances carbon and nitrogen content, leading to changes in soil physical and chemical properties and influencing above-ground plant diversity. These factors contribute to variations in soil redox conditions, ultimately affecting the quantitative and structural dynamics of soil microbial communities (53).

The results also reveal the response of underground networks to different topographic habitats. Soil bacterial and fungal networks exhibit distinct properties and respond differently to terrain. The effect of topographic habitat on fungal networks is greater than on bacterial networks, consistent with previous research (54, 55). The structural characteristics of soil microbial communities may be influenced by the biological interactions and relationships among species and environmental factors (such as topography, light, soil, and woody plants (56–58)). Although the relationships between soil microorganisms are complex (59, 60), changes in species abundance may affect the stability of these networks and consequently, the response of soil microbial communities to terrain. Thus, ecological specialization plays a crucial role in the distribution of soil microorganisms.

### Effects of topographic habitat on the spatial distribution of soil microorganisms

The Torus transformation test showed that 59.99% of fungal OTUs and 42.72% of bacterial OTUs were related to at least one terrain habitat. This finding indicates that different soil microorganisms exhibit dissimilar distribution preferences in different terrain habitats. A likely reason is that fungi are more specialized than bacteria, and bacteria have high intrinsic growth rates and single-cell characteristics, so bacteria are more resilient than fungi in the face of environmental changes (16). In terms of biological factors, significant differences are noted in soil microbial richness and species composition in different terrain habitats, and species competition (61) and species succession (62, 63) also affect the distribution of soil microorganisms in forest ecosystems. In terms of abiotic environment, topographic factors (56, 57, 64), light (65, 66), soil (67), and abiotic factors (68) can affect soil physical and chemical properties and nutrients and influence the distribution of soil microorganisms. Therefore, different soil microorganisms show dissimilar distribution preferences in diverse topographic habitats of temperate deciduous broadleaf forests.

### Effects of environmental factors on soil microbial communities

Niche theory emphasizes that species composition is closely related to changes in environmental factors (69). Some studies support niche partitioning and confirm that abiotic factors influence the composition of microbial communities (70, 71). Therefore, environmental heterogeneity is a key factor in exploring the distribution of soil microorganisms. Although ecologists have conducted a series of explorations (15, 72), the main environmental driving forces of soil microbial distribution in different terrain habitats are still unclear. In our study, environmental factors affect the distribution of soil microorganisms. Soil physical and chemical factors and topographic factors play a vital role in the distribution of soil microorganisms (73–76). SWC, pH, SOM, N, slope, and elevation have a greater effect on the distribution of bacterial communities and fungal communities (Fig. 6A through C; Table 1). SWC and pH are key factors affecting the microbial assembly process in forest ecosystems (77, 78). Soil pH may affect the distribution of soil microorganisms by influencing the adaptation and survival of soil microorganisms (78). The importance of N to the distribution of soil microorganisms may be related to the suitability of soil microorganisms under different inorganic nitrogen concentrations (79), and soil organic matter affects the survival of soil microorganisms by affecting soil physical and chemical properties and soil matrix composition (80). Soil physical and chemical factors explain bacterial communities more than fungal communities possibly because bacteria have a wider niche width and higher growth and turnover rates, responding more quickly and evidently to changes in the soil environment. Therefore, the different responses of fungi and bacteria to environmental factors affect the distribution pattern of soil microorganisms.

### Conclusion

In this paper, the structural composition and spatial distribution characteristics of soil microorganisms in different terrain habitats of the temperate deciduous broad-leaved forest were examined. The differences in the composition and structure of soil microbial communities primarily stem from terrain habitats. Fungi were distributed in valleys with high moisture content, whereas bacteria were less sensitive to terrain changes, and fungi showed a high degree of specialization. In addition, soil physical and chemical properties (pH, SWC, SOM, and N) explained the changes in bacterial community distribution more than fungal communities, and SWC had a greater effect on fungi than on bacteria.

The results are important for understanding how complex soil microbial communities respond to different terrain habitats. In sustainable forest management, differentiated management strategies should be implemented according to different terrain habitat characteristics to maintain the diversity and ecological specialization of microbial communities, thereby improving soil resilience and productivity and increasing the sustainability of forest ecosystems.

## Data Availability

The raw reads of sequencing data are available in the NCBI BioProject SRA database under accession number PRJNA785719.

## SUPPLEMENTAL MATERIAL

Supplemental material spectrum.02056-24-s0001.docx Fig. S1 and S2.

ASM does not own the copyrights to Supplemental Material that may be linked to, or accessed through, an article. The authors have granted ASM a non-exclusive, world-wide license to publish the Supplemental Material files. Please contact the corresponding author directly for reuse.
