# Supplementary material for: Soil microorganism distributions depend on habitat partitioning of topography in a temperate mountain forest
Source: Microbiol Spectr. 2025 May 28;13(7):e02056-24. doi: 10.1128/spectrum.02056-24 (PMC12210923; doi:10.1128/spectrum.02056-24)
Supplement: Supplemental material — Fig. S1 and S2. [file spectrum.02056-24-s0001.docx]

Appendix


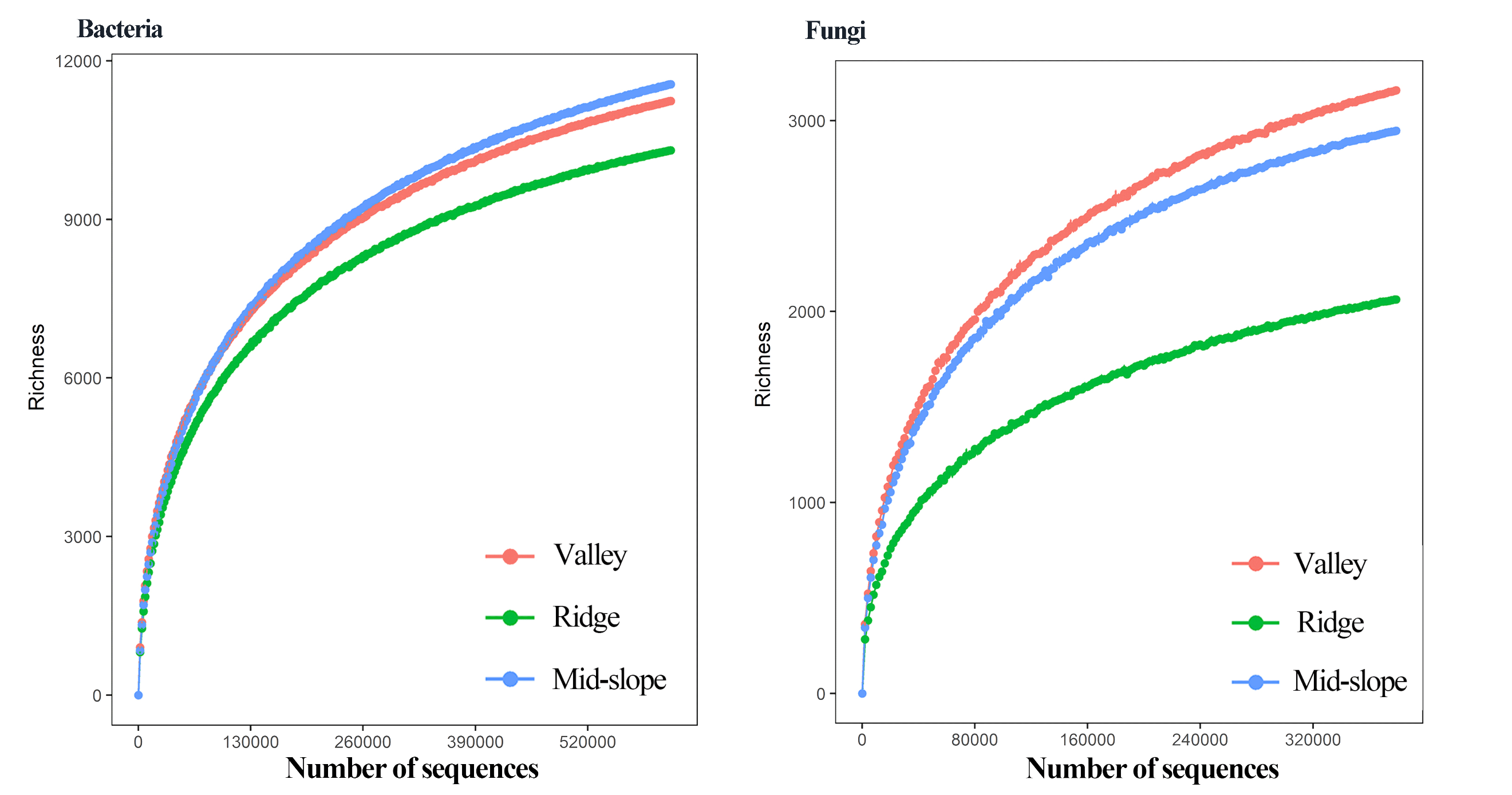


S 1. Rarefaction curves showing the observed OTU richness after rarefaction for all samples across three different terrain habitat communities


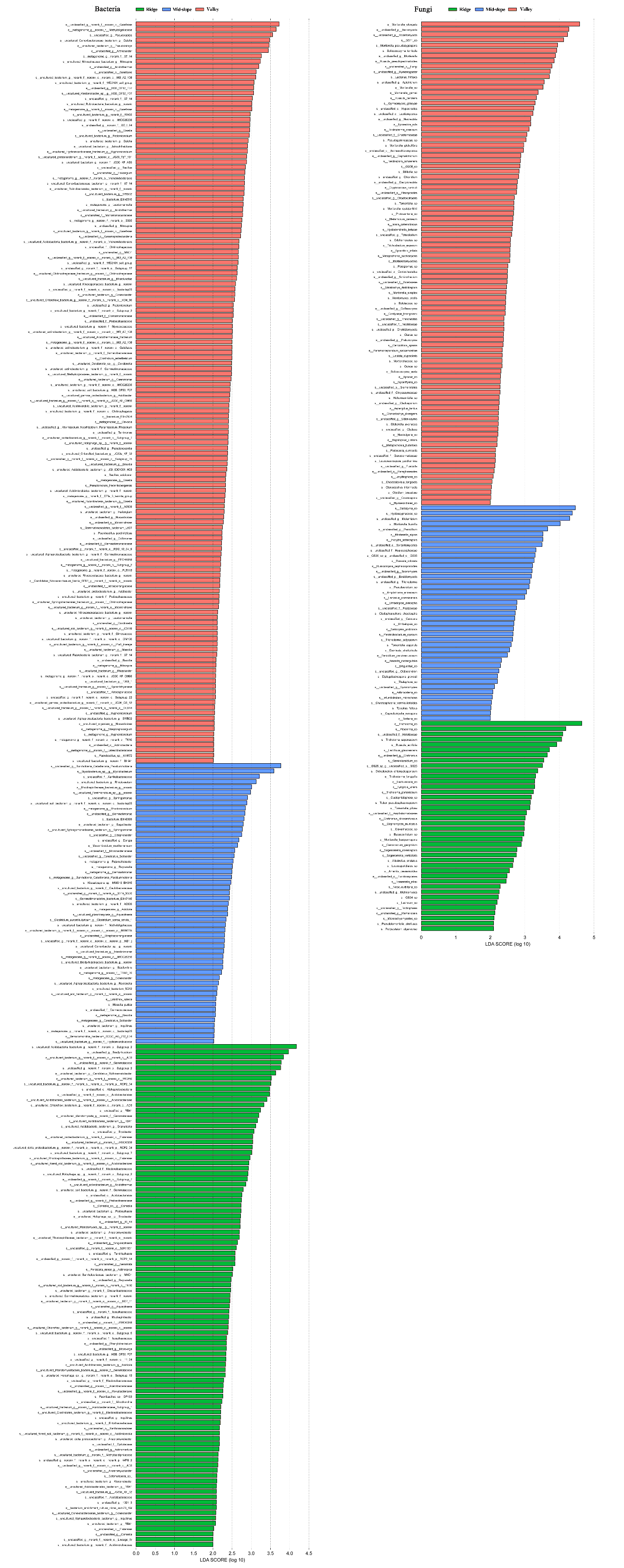


S 2. Histogram of bacteria and fungi analyzed based on LEfSe, with a threshold of 2. The red, green, and blue bars represent soil microorganisms with significant differences in the three terrain habitats. The length of the bar represents the effect size of the significantly distinct species. The longer the bar, the more significant the difference.
